# First-Principles Study on the Modulation of Schottky Barrier in Graphene/Janus MoSSe Heterojunctions by Interface Contact and Electric Field Effects

**DOI:** 10.3390/nano15151174

**Published:** 2025-07-30

**Authors:** Zhe Zhang, Jiahui Li, Xiaopei Xu, Guodong Shi

**Affiliations:** School of Physics and Advanced Energy, Henan University of Technology, Zhengzhou 450001, China; 2024931246@stu.haut.edu.cn (J.L.);

**Keywords:** Gr/Janus MoSSe heterojunction, Schottky-to-Ohmic transition, electric field modulation, Interface charge transfer, two-dimensional materials

## Abstract

Constructing heterojunctions can combine the superior performance of different two-dimensional (2D) materials and eliminate the drawbacks of a single material, and modulating heterojunctions can enhance the capability and extend the application field. Here, we investigate the physical properties of the heterojunctions formed by the contact of different atom planes of Janus MoSSe (JMoSSe) and graphene (Gr), and regulate the Schottky barrier of the Gr/JMoSSe heterojunction by the number of layers and the electric field. Due to the difference in atomic electronegativity and surface work function (WF), the Gr/JSMoSe heterojunction formed by the contact of S atoms with Gr exhibits an n-type Schottky barrier, whereas the Gr/JSeMoS heterojunction formed by the contact of the Se atoms with Gr reveals a p-type Schottky barrier. Increasing the number of layers of JMoSSe allows the Gr/JMoSSe heterojunction to achieve the transition from Schottky contact to Ohmic contact. Moreover, under the control of an external electric field, the Gr/JMoSSe heterojunction can realize the transition among n-type Schottky barrier, p-type Schottky barrier, and Ohmic contact. The physical mechanism of the layer number and electric field modulation effect is analyzed in detail by the change in the interface electron charge transfer. Our results will contribute to the design and application of nanoelectronics and optoelectronic devices based on Gr/JMoSSe heterojunctions in the future.

## 1. Introduction

Graphene (Gr), first synthesized experimentally in 2004 [1], has excellent properties such as high carrier mobility, prominent mechanical strength, attractive quantum Hall effect [2], and massless Dirac fermion [3]. Moreover, proton transport and hydrogenation in graphene-based molecular devices can be modulated through dual-gate control [4]. It is one of the typical representatives of the 2D materials family, which has aroused great interest in the past decade. Although Gr is a promising material for the design of nanoelectronic devices in the future [5], its photoelectric application is limited due to the lack of bandgap and fast carrier recombination. Two-dimensional transition metal dichalcogenides (TMDCs) MX_2_ (M = Mo, W; X = S, Se) exhibit remarkable performance in high-end optoelectronic and electronic devices [6,7,8]. The optical and electronic characters of TMDCs can be effectively controlled by managing the composition of the alloy [9,10,11]. In addition, the 2H phase JMoSSe can be obtained either by completely replacing the top-layer sulfur atoms in a MoS_2_ monolayer with selenium atoms [12], or by replacing the top-layer selenium atoms in a MoSe_2_ monolayer with sulfur atoms [13]. Compared to 2D MX_2_ with conventional mirror symmetry, JMoSSe has mirror asymmetry, showing out-of-plane piezoelectricity [14] and anomalous photovoltaics [15], which has important application prospects in nano piezoelectric devices and photovoltaics.

In order to integrate the superior property of different 2D materials and to eliminate the disadvantages of a single material, constructing heterojunctions and adopting various modulation methods can be used to improve the performance of 2D materials and expand their application field [16,17,18]. Vertical heterojunctions based on 2D van der Waals (vdW) materials have absorbed a great deal of interest. For example, the formation of a heterojunction by combining Gr with other 2D materials is one of the tactics to enlarge its application. A variety of 2D ultra-thin Gr-based vdW heterojunctions, such as Gr/GaSe [19], Gr/h-BN [20,21], Gr/MoS_2_ [22,23], and Gr/phosphorene [24,25], have been widely explored both theoretically and experimentally. Specifically, the devices based on Gr/h-BN have the potential of stable and efficient electro–optical signal conversion in photon interconnection [21]. The Gr/MoS_2_ heterojunction is used in memory and electronics devices with an on/off current ratio of up to 100 [22,23]. The excellent electrochemical properties of the sandwich type Gr/phosphorene hybrid make it an appropriate anode material for sodium-ion batteries [24]. These excellent performances make Gr-based vdW heterojunctions attractive candidates for nanoelectronic devices. However, for these newfangled microelectronic devices based on vdW heterojunctions, the contact interface between Gr and 2D semiconductor materials will form a Schottky barrier, which has a crucial impact on the performance of the device. In general, low Schottky barrier height (SBH) signifies low contact resistance and high electronic injection efficiency. Therefore, many studies have been carried out to regulate the Schottky barrier of these Gr-based vdW heterojunctions. The Schottky barrier can be modulated by the number of layers [26,27], interlayer distance [28,29], electric field [30,31,32], and so forth. For instance, the bandgap of the layered semiconductor decreases as the number of layers increases [33,34], and increasing the number of layers of InSe causes a transition from a Schottky contact to an Ohmic contact [26]. The contact properties of the Gr/SnS heterojunction can be effectively adjusted by an external electric field, which can cause a transition from a p-type to an n-type Schottky contact and then to an Ohmic contact [30].

Motivated by these exciting achievements in the construction of Gr-based vdW heterojunctions and the regulation of Schottky barriers, as well as the novel properties exhibited by JMoSSe owning to structural asymmetry, it is worthwhile to investigate whether Gr/JMoSSe heterojunctions have peculiar physical properties and whether they have superior performance in the design and application of nanoelectronic devices. In this work, the electronic band structure and Schottky barrier of Gr/JSMoSe heterojunction in S-C atom contact and Gr/JSeMoS heterojunction in Se-C atom contact are studied by first-principles calculations. Our results show that the heterojunctions of two different contact interfaces exhibit different types of Schottky barriers, whereas the electronic band structure properties of the individual JMoSSe and Gr are well preserved in the Gr/JMoSSe heterojunction. The number of layers of MoSSe can regulate the SBH of the Gr/JMoSSe heterojunction and achieve a transition from a Schottky contact to an Ohmic contact as it is added to the third layer. In addition, the external vertical electric field can also effectively control the SBH of the Gr/JMoSSe heterojunction and realize the transformation of p-type Schottky barrier, n-type Schottky barrier, and Ohmic contact. Furthermore, in order to obtain insight into the physical mechanism of layer number and electric field regulation, we analyzed the number of interface electron charge transfer in detail through the Mulliken population and explained the relationship between Schottky barrier change and the number of electron charge transfers. Our results are of great significance to understand the fundamental physical properties of metal–semiconductor contact and the application of new nanoelectronic devices in the future.

## 2. Computational Models and Method

**Figure 1:**
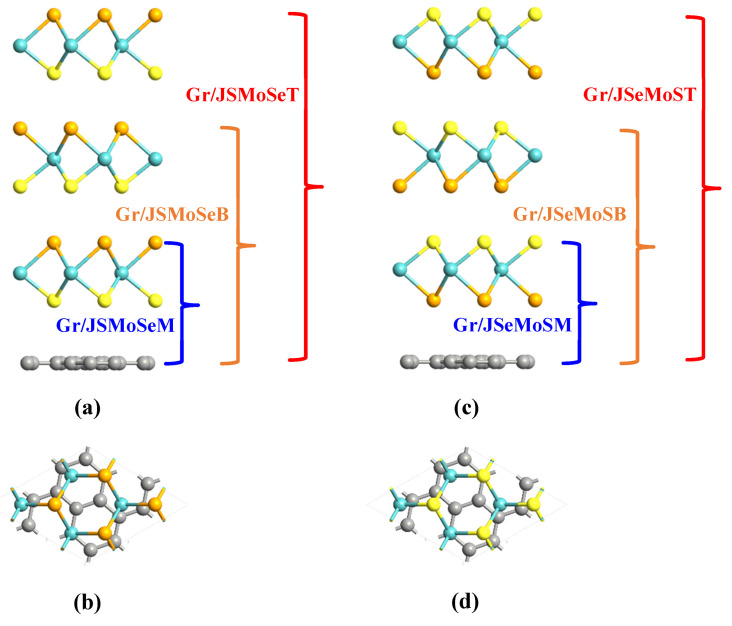
The side and top view of the Gr/JSMoSe heterojunctions are shown in (**a**,**b**). (**c**,**d**) exhibit the side and top view of the Gr/JSeMoS heterojunctions. Blue, orange, and red correspond to Gr/JSMoSeM (Gr/JSeMoSM), Gr/JSMoSeB (Gr/JSeMoSB), and Gr/JSMoSeT (Gr/JSeMoST) heterojunctions, respectively.

The bulk graphite is a layered hexagonal structure belonging to space group 194 and the lattice constants of bulk graphite are $a_{G}=b_{G}=2.4612$ and $c_{G}=6.709$ Å. The monolayer Gr that is optimized from bulk graphite has lattice constants of $a_{Gr}=b_{Gr}=2.4566$ Å, which are consistent with the experimental measurement [35] and theoretical values [36,37]. The in-plane lattice constants obtained by geometric optimization of JMoSSe monolayer are $a_{J}=b_{J}=3.2489$ Å, which is in good agreement with experimental and theoretical results [12,13]. In order to search for the equilibrium structure between finding the minimum number supercell atoms and the minimum lattice mismatch, $\sqrt{7}\times \sqrt{7}$ supercell of Gr monolayer and 2×2 supercell of JMoSSe monolayer are used to form a Gr/JMoSSe heterojunction. The mean absolute strain induced by lattice mismatch is 0.02% when the Gr and the monolayer JMoSSe combine to form a heterojunction, which will maintain the excellent intrinsic physical properties of Gr and JMoSSe as much as possible. When Gr and JMoSSe are in contact to form a heterojunction, two kinds of interfaces are formed; one is C atoms that are in contact with the S atomic layer, and the other is C atoms that are in contact with the Se atomic layer. Figure 1a,b shows the side and top views of Gr/JSMoSe heterojunction. Blue, orange, and red correspond to the Gr/JSMoSeM, Gr/JSMoSeB, and Gr/JSMoSeT heterojunction formed by the Gr and JMoSSe monolayer, bilayers, and trilayers, respectively. Figure 1c,d displays the side and top views of Gr/JSeMoS heterojunction. Blue, orange, and red correspond to the Gr/JSeMoSM, Gr/JSeMoSB, and Gr/JSeMoST heterojunction, respectively.

Our calculations of total energy, electronic band structure, and other physical properties in the Gr/JMoSSe heterojunction are performed by density functional theory (DFT) [38], which is achieved in the QuantumATK [39]. The QuantumATK in association with linear combination of atomic orbitals (LCAO) method within the generalized gradient approximations (GGA) of Perdew–Burke–Ernzerhof (PBE) exchange–correlation functional is used [40]. The electron wave function is extended using the SG15 [41,42,43] pseudopotential and the basis set is selected to high accuracy. The density mesh cutoff energy was chosen to be 370 Ry for all calculations. The Monkhorst–Pack κ-point grid [44] of 8×8×1 is applied to the calculations for $\sqrt{7}\times \sqrt{7}$ supercell of monolayer Gr, 2×2 supercell of monolayer JMoSSe, and Gr/JMoSSe heterojunction, respectively, which can ensure that all three have the same sampling density in the reciprocal space. The vdW interaction will be contained through the semi-empirical Grimme [45] correction which does not attempt to portray the actual source of the interaction (fluctuating dipoles) but rather its impact on the DFT mean-field effective potential. The DFT-D2 functional developed by Grimme [45] aggrandizes an attached section to the DFT total energy for purpose of accounting for vdW interaction, or dispersion forces. However, if we perform Grimme correction but do not consider the basis set superposition error (BSSE) caused by incompleteness of the LCAO basis set, the calculated interlayer spacing between Gr and JMoSSe will not be accurate enough. Therefore, considering the van der Waals interaction in the Gr/JMoSSe heterojunction and the resulting BSSE of LCAO basis sets, the Grimme DFT-D2 semi-empirical correction [45] and counterpoise correction [46] are taken into account in the QuantumATK software (2022.12) to accurately calculate the physical properties of the Gr/JMoSSe heterojunction. The periodic boundary condition was employed to simulate the supercell of Gr, JMoSSe, and Gr/JMoSSe heterojunction. The layered 2D atomic surface is set on the *x-y* coordinate plane and a vacuum thickness of 18 Å is applied to restrain interaction between periodic images of slabs in the *z* direction. The Limited-memory Broyden–Fletcher–Goldfarb–Shanno (LBFGS) algorithm [47] is applied to optimize all the geometric structures. The geometrical optimization is used to relax the ion position and unit cell volume with the force tolerance and stress error tolerance converged to 0.01 eV/Å and 0.001 eV/Å^3^, respectively. When calculating band structures, 100 points are collected between every two high-symmetry points in reciprocal space. We choose to demonstrate 20 points in the graph and set the Fermi level ($E_{F}$) to zero in the band structures.

## 3. Results and Discussion

### 3.1. Van der Waals Stacking Modulation of Schottky Barrier of Gr/JMoSSe Heterojunctions

Table 1 lists the equilibrium interlayer distance ($d_{\mathrm{eq}}$), which is defined as the average distance between the nearest neighboring atom layers. The average binding energy ($E_{b}$) can be defined as

(1) $$E_{b}=\left(E_{H}-E_{G}-E_{J}\right)/I$$

where $E_{H}$, $E_{G}$, and $E_{J}$ represent the total energy of Gr/JMoSSe heterojunction, monolayer Gr, and JMoSSe, respectively, and I is the area of the contact interface between Gr and JMoSSe. The WF is defined as the difference between the vacuum level and the $E_{F}$. Based on the Schottky–Mott rules for metal–semiconductor interfaces [48], the n-type SBH ($\Phi_{\mathrm{Bn}}$) is measured by the energy difference between the conduction band minimum ($E_{C}$) of the semiconducting layer and the $E_{F}$ ($\Phi_{\mathrm{Bn}}=E_{C}-E_{F}$), and the p-type SBH ($\Phi_{\mathrm{Bp}}$) is defined as the energy difference between the $E_{F}$ and the valence band maximum ($E_{V}$) of the semiconducting layer ($\Phi_{\mathrm{Bp}}=E_{F}-E_{V}$). When SBH becomes negative, the Schottky contact turns into an Ohmic contact. The sum of the n-type barrier height and the p-type barrier height is approximately equal to the bandgap ($E_{g}$) of the semiconductor. The equilibrium interlayer distance and average binding energy of Gr and JMoSSe correspond to the case of bilayer stacking. The interlayer distance of the bilayer Gr is 3.36 Å and the WF of monolayer Gr is 4.46 eV, which is consistent with the reported results [35,36]. The equilibrium interlayer distances of the Gr/JMoSSe heterojunction represent the average distance between Gr monolayer and JSMoSeM, JSMoSeB, JSMoSeT, JSeMoSM, JSeMoSB, and JSeMoST, respectively.

**Figure 2:**
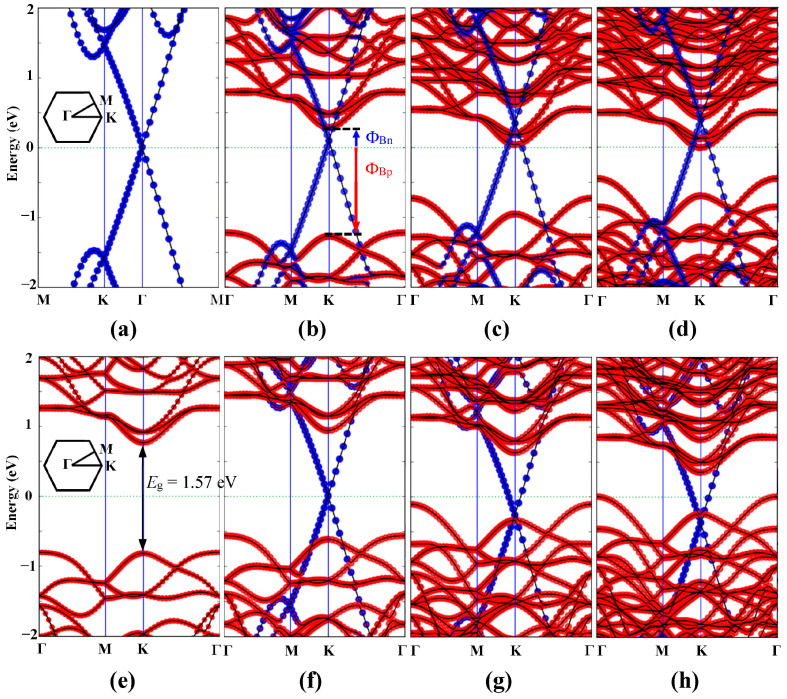
The projected band structure of (**a**) $\sqrt{7}\times \sqrt{7}$ supercell of Gr monolayer, (**b**) Gr/JSMoSeM heterojunction, (**c**) Gr/JSMoSeB heterojunction, (**d**) Gr/JSMoSeT heterojunction, (**e**) 2×2 supercell of Janus MoSSe monolayer, (**f**) Gr/JSeMoSM heterojunction, (**g**) Gr/JSeMoSB heterojunction, and (**h**) Gr/JSeMoST heterojunction, respectively.

As shown in Table 1, the interlayer distances range from 3.25 to 3.48 Å, which are much larger than the total of the covalent radii between the interface atoms, indicating that vdW coupling dominates the interlayer interaction. The calculated binding energy is between −23.46 and −29.78 meV/Å^2^, which is lower than the binding energy of the typical range of the vdW layered interaction (−21~−13 meV/Å^2^) [49], meaning that the structures we studied are more stable. The fourth column of data in Table 1 represents the WF of the Gr monolayer, the JMoSSe monolayer, and the JMoSSe covering the Gr surface, respectively. The JMoSSe has two different surfaces, which correspond to different WFs. The WF of the surface of the Se atoms is 4.46 eV, whereas the surface of the S atom has a WF of 5.24 eV. For the Gr/JSMoSe heterojunction, the WF decreases as the number of layers increases, indicating that the $E_{F}$ is shifted up. Conversely, the WF of the Gr/JSeMoS heterojunction increases as the number of layers increase, which causes the $E_{F}$ to move down. These results can be verified by the projected band structures in Figure 2. The reason for this interesting physical phenomenon is that the difference in the electronegativity of asymmetric atoms causes the space charge to accumulate at different locations, resulting in different magnitudes and directions of the dipole moments. That is, adding different interfaces will cause the $E_{F}$ to move in different directions. The SBH of the Gr/JMoSSe heterojunction will also change accordingly. As exhibited in the last column of Table 1, Due to the different contact interfaces, the Gr/JSMoSe heterojunction and the Gr/JSeMoS heterojunction exhibit different Schottky barrier heights and types. The results calculated by Chen et al. using the k-projection method show that the interface interactions between Gr-bilayer and the two surfaces of SiC (0001) behave differently [50]. However, for the Gr/JSMoSe and the Gr/JSeMoS heterojunction, the outcome is that the heterojunctions will achieve a transition from Schottky contact to Ohmic contact when added to the third layer.

It is well known that SBH modulation is very significant in electronic device applications [51]. In general, increasing the number of layers is a feasible method to effectively regulate the SBH of the Gr-based vdW heterojunctions [25,27]. Therefore, we explored the effect of the number of layers on the SBH and electronic band structure of G/JMoSSe. The projected band structures of G/JMoSSe with different layers are displayed in Figure 2. The massless Dirac cone in Figure 2a is located at the Γ high symmetry point, which perfectly preserves the excellent electronic band structure properties of Gr. The band structure of the JMoSSe monolayer shows a direct bandgap of 1.57 eV in Figure 2e, which agrees well with theoretical calculations and experimental values [12,13]. As shown in Figure 2b, there is an n-type SBH of 0.28 eV that is formed when Gr and the S atomic surface are combined to form the Gr/JSMoSeM heterojunction. Since the WF of the surface of the S atomic layer is larger than that of Gr, the $E_{F}$ of the Gr/JSMoSe heterojunction is slightly lower than Dirac cone of the Gr. Figure 2f depicts that a p-type SBH of 0.61 eV is formed when Gr and the Se atomic surface are combined to form the Gr/JSeMoSM heterojunction. Meanwhile, due to the same WF at the interface, the Dirac cone of Gr is located at the $E_{F}$ of Gr/JSeMoSM heterojunction. As described in Figure 2c,d, the $E_{F}$ of the Gr/JSeMoS heterojunction moves upward as the number of layers increases, and the n-type SBH decreases. Figure 2g,h show the opposite result; the $E_{F}$ of Gr/JSMoSe heterojunction moves down as the number of layers increases, and the p-type SBH decreases. The same phenomenon is that the Ohmic contact replaces the Schottky contact when added to the third layer. This is due to the difference in electronegativity of the atoms, resulting in space charge transfer and redistribution [30], forming interface dipoles [20,27], which in turn causes the $E_{F}$ to relatively move to control the SBH. Specifically, the order of atomic electronegativity is 2.50 (C) > 2.48 (Se) > 2.44 (S) > 1.30 (Mo) [52], which indicates the direction of electron transfer. The amount of charge transfer between atoms can be accurately quantified by the Mulliken population [53]. The total number of electrons, N, is given by

(2) $$N=\sum_{ij}D_{ij}S_{ij}$$

where S is the overlap matrix and D is the density matrix. The Mulliken population can accurately obtain the amount of charge distribution on each orbit and atom. The Mulliken population of orbitals is

(3) $$M_{i}=\sum_{j}D_{ij}S_{ji}$$

where $M_{i}$ is defined by restricting one of the sum indexes to the orbital. The Mulliken population of atoms is

(4) $$M_{\mu }=\sum_{i\in \mu }\sum_{j}D_{ij}S_{ji}$$

where $M_{\mu }$ is defined by adding up all the orbital contributions on atom number μ. For the JMoSSe monolayer we studied, the charge transfer amounts of S, Mo, and Se atoms were 0.1720, −0.5230, and 0.3510 e/atom, respectively. A positive value is the amount of electron charge obtained, and a negative value corresponds to the amount of electron charge lost. When C and S atoms are combined to form the Gr/JSMoSeM heterojunction, the charge transfer amounts of C, S, Mo, and Se atoms are 0.0095, 0.2213, −0.6188, and 0.3648 e/atom, respectively. In the Gr/JSeMoSM heterojunction of C atoms interacting with Se atoms, the charge transfer amounts of C, Se, Mo, and S atoms are 0.0084, 0.2410, −0.4470, and 0.1778 e/atom, respectively. The difference in interlayer spacing between Gr/JSMoSeM and Gr/JSeMoSM is very small, and the resulting impact can be ignored. The reason why the Schottky barriers of Gr/JSMoSeM and Gr/JSeMoSM are different is that the electronegativity of S and Se are different. Differences in atomic electronegativity lead to differences in WF and space charge distribution. For the Gr/JSMoSe heterojunctions, the amount of charge obtained by C atoms gradually decreases as the number of JMoSSe layers increase (the amount of charge obtained by the C atoms in Gr/JSMoSeM, Gr/JSMoSeB, and Gr/JSMoSeT heterojunctions is 0.0095, 0.0094, and 0.0066 e/atom, respectively.), which causes the $E_{F}$ originally located in the Dirac cone to move down. The observed asymmetric trend in charge transfer with layer number reflects the intrinsic dipole orientation of Janus MoSSe, where stacking modulates the electrostatic potential across the heterojunction in a termination-dependent manner. As shown in Figure 2b–d, a decrease in the amount of charge on the C atoms means that the amount of charge on JMoSSe increases, which causes the $E_{F}$ to move up and gradually approach the $E_{C}$ of JMoSSe. However, the amount of charge obtained by the C atoms in Gr/JSeMoSM, Gr/JSeMoSB, and Gr/JSeMoST heterojunctions are 0.0084, 0.0087, and 0.0099 e/atom, respectively. The charge obtained by the C atoms increases as the number of JMoSSe layers increases, which causes the Fermi energy initially located in the Dirac cone to move up. An increase in the charge on the C atoms implies that the charge on JMoSSe is reduced. As shown in Figure 2f–h, the electron reduction in JMoSSe causes the $E_{F}$ to move downward and gradually contact $E_{V}$ of JMoSSe. Therefore, increasing the number of semiconductor layers can effectively adjust the SBH and achieve the transition of Ohmic contact.

**Figure 3:**
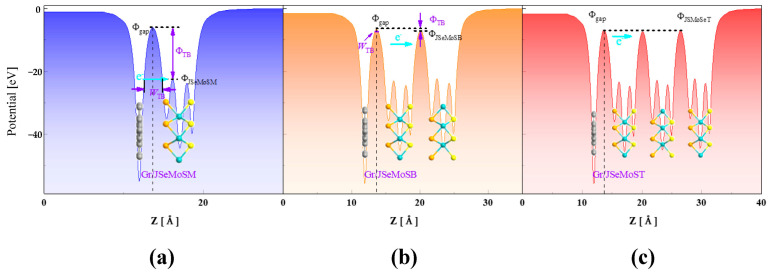
The average effective potential profile of (**a**) Gr/JSeMoSM, (**b**) Gr/JSeMoSB, and (**c**) Gr/JSeMoST heterojunctions, respectively. The vacuum energy level is specified to be zero.

The number of layers of the semiconductor can also modulate the metal-semiconductor’s tunneling barrier, which is an important feature reflecting the semiconductor-metal contact performance and is characterized by the height and width of the tunneling barrier [54,55,56]. Since the 2D material is bonded at the interface by vdW interaction without strong orbital overlap, the tunnel barrier is always present in the vdW coupling contact. In order to improve the interface current in the vdW interface contact, the tunneling barrier should be small enough to promote the carrier transmission probability. Narrow barrier width and low barrier height mean low contact resistance and high electronic injection efficiency. The size of the tunneling barrier can be obtained by calculating the effective potential [26,54,56]. As clarified in Figure 3, the tunneling barrier height ($\Phi_{\mathrm{TB}}$) is defined as the difference between the vdW gap ($\Phi_{\mathrm{gap}}$) and the potential energy of JMoSSe ($\Phi_{\mathrm{JMoSSe}}$). The width of the square potential barrier is employed as the tunneling barrier width ($W_{\mathrm{TB}}$). The tunneling possibility ($P_{\mathrm{TB}}$) is calculated according to the following equation [26]:

(5) $$P_{\mathrm{TB}}=\exp\left(-\frac{2W_{\mathrm{TB}}}{\hbar }\sqrt{2m\Phi_{\mathrm{TB}}}\right)$$

where *m* is the mass of the free electron and *ħ* is the reduced Planck’s constant. It can be seen from Figure 3a that the Gr/JSeMoSM heterojunction has a large barrier height and width, and the probability of electron tunneling is $9.22\times 10^{-5}$, which means that electrons cannot substantially pass through the barrier. However, when the number of layers is increased to two layers in Figure 3b, the barrier height and width are significantly reduced, and the possibility of tunneling has reached 61%. This shows that the number of layers has a significant modulation effect on the barrier. Furthermore, the barrier of the Gr/JSeMoST heterojunction is shown to disappear substantially in Figure 3c, and electrons transport through the interface is facilitated by thermal excitation.

### 3.2. The Regulating Effect of Vertical Electric Field on the Schottky Barrier of Gr/JMoSSe

**Figure 4:**
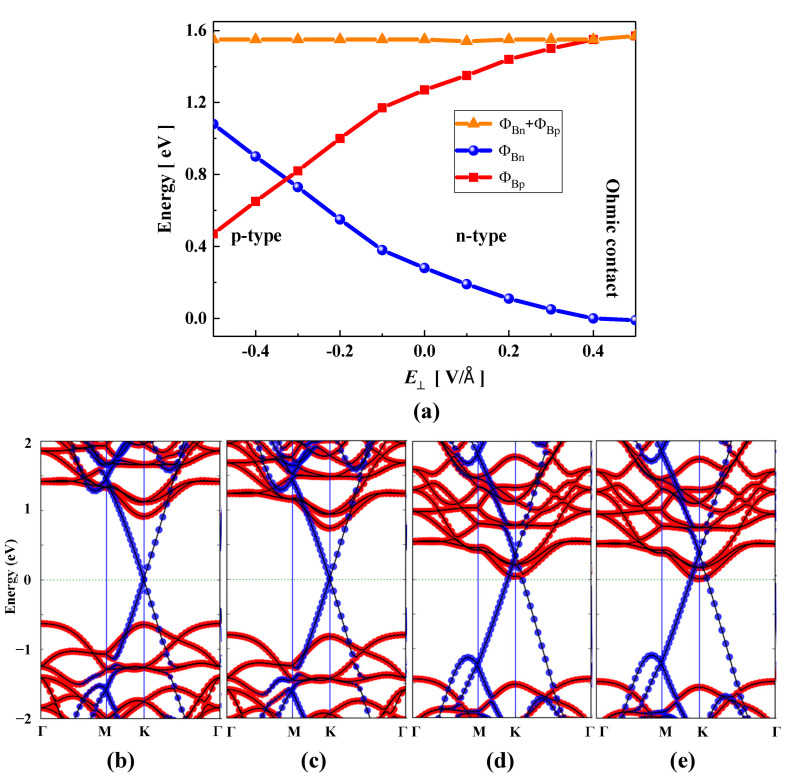
(**a**) Schottky barrier variations in Gr/JSMoSeM heterojunction as a function of vertical electric field. The orange, red, and blue regions represent p-type Schottky contacts, n-type Schottky contacts, and Ohmic contacts, respectively. The projected band structure of Gr/JSeMoSM heterojunction at the electric field strength of (**b**) −0.4, (**c**) −0.3, (**d**) 0.3, (**e**) 0.4 V/Å, respectively.

The external vertical electric field (*E*_⊥_) effect plays an important role in the high performance of Gr-based vdW FET devices [57]. Therefore, the regulation of the electric field on the Schottky barrier is crucial for the application of Gr-based devices in the field of microelectronics. In our work, we studied the effect of a vertical electric field on the Schottky barrier of Gr/JMoSSe heterojunction with different layers. The direction with JMoSSe (Gr) pointing to Gr (JMoSSe) is defined as the electric field in the positive (negative) direction. Figure 4a shows the variation in the Schottky barrier as a function of the vertical electric field. It can be found that the positive electric field reduces n-type SBH and increases p-type SBH. When the electric field strength is increased to 0.4 V/Å, the Schottky barrier height effectively vanishes, indicating a transition to Ohmic contact behavior. This result originates from the band alignment of the Gr/JSMoSeM heterojunction, which shows that the conduction band minimum (CBM) of MoSSe intersects with the Fermi level. When a negative electric field is applied, the n-type and p-type SBH have opposite trends. The n-type Schottky barrier transitions to a p-type Schottky barrier when the electric field strength reaches −0.33 V/Å. In order to fully understand the physical origin of the relationship between the SBH and the electric field strength, we studied the evolution of the projected band structure and the electron charge transfer mechanism under the electric field in detail. In Figure 4d,e, as the positive electric field strength increases, the $E_{F}$ of Gr/JSMoSeM heterojunction moves up until it reaches the $E_{C}$ of JSMoSeM, achieving a transition of the n-type Schottky contact to the Ohmic contact. Interestingly, The Dirac cone of Gr moves up relative to the $E_{F}$ under a positive electric field. In other words, the $E_{F}$ of Gr moves downwards relative to the Dirac cone under a positive electric field. In contrast, as shown in Figure 4b,c, the $E_{F}$ of Gr/JSMoSeM heterojunction moves downwards to approach the $E_{V}$ of JSMoSeM under a negative electric field. The amount of charge transfer obtained from the calculation of the Mulliken population can be used to reveal its most fundamental physical principle. For Gr in the Gr/JSMoSeM heterojunction, the electron charge obtained for each C atom is 0.0115, 0.0112, 0.0095, 0.0079, and 0.0065 e at the electric field intensity of −0.4, −0.3, 0, 0.3, and 0.4 V/Å, respectively. The positive electric field reduces the amount of electron charge on the C atom, whereas the negative electric field increases the amount of electron charge on the C atom. The increase in the amount of electron charge on the C atom makes the $E_{F}$ to move upward relative to the Dirac cone, and the decrease in the amount of electron charge causes the $E_{F}$ to move downward relative to the Dirac cone. In the Gr/JSMoSeM heterojunction, an increase in the amount of electron charge of Gr suggests a decrease in the amount of electron charge of JSMoSeM, and vice versa. Therefore, the downward movement of the $E_{F}$ relative to the Dirac cone of Gr means that the $E_{F}$ moves upward with respect to the $E_{C}$ of JSMoSeM moves upward, and vice versa. In short, the electric field can convert the Schottky contact to an Ohmic contact, or the n-type Schottky barrier can be converted to a p-type Schottky barrier. The intrinsic physical mechanism is that the electric field changes the space charge distribution to reconstruct the space charge, causing the relative movement of the $E_{F}$.

**Figure 5:**
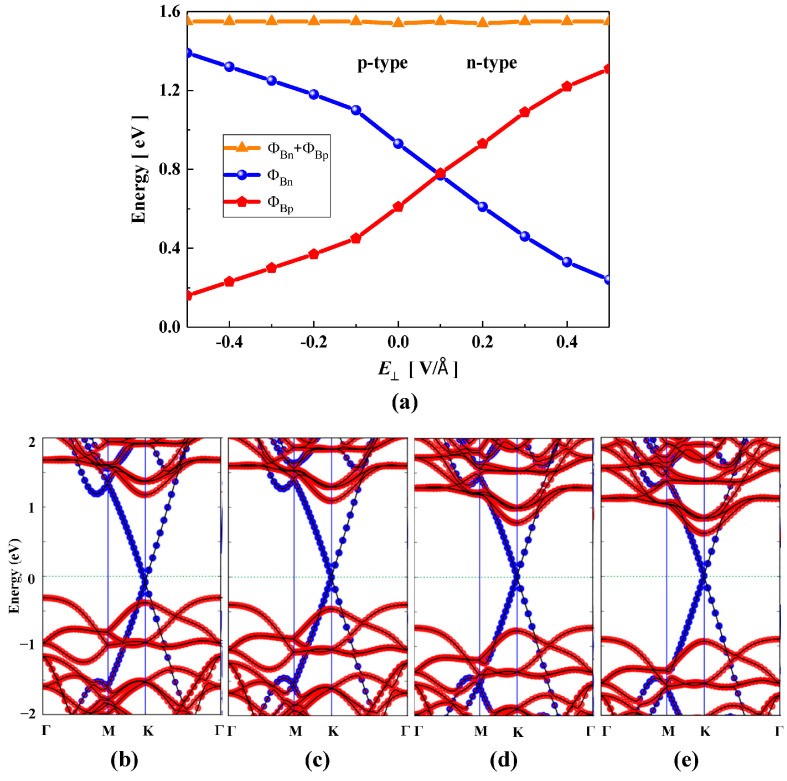
(**a**) Schottky barrier variations in Gr/JSeMoSM heterojunction as a function of vertical electric field. The projected band structure of Gr/JSMoSeM heterojunction when the vertical electric field strength is (**b**) −0.2, (**c**) −0.1, (**d**) 0.1, (**e**) 0.2 V/Å, respectively.

Figure 5a depicts the effect of the electric field on the Schottky barrier of Gr/JSeMoSM heterojunction. It can be found that the n-type and p-type SBHs have opposite trends under the electric field. Specifically, the n-type SBH decreases under a positive electric field and increases under a negative electric field. The p-type SBH changes exactly the opposite. The transition from the p-type to the n-type Schottky barrier occurs when the electric field strength reaches 0.1 V/Å. The variation in the SBH can be elucidated by the evolution of the projected band structure. Figure 5b–e depicts the projected band structures of Gr/JSeMoSM heterojunction at the electric field strength of −0.2, −0.1, 0.1, and 0.2 V/Å, respectively. During the change from negative electric field to positive electric field, the $E_{C}$ of JSeMoSM approaches the $E_{F}$, whereas the $E_{V}$ is opposite. The $E_{F}$ of Gr is also shifted up relative to the Dirac cone. This is because, as mentioned before, the external electric field changes the distribution of space charge in the Gr/JSeMoSM heterojunction and causes the relative shift in the $E_{F}$. Specifically, for Gr in the Gr/JSeMoSM heterojunction, the electron charge obtained for each C atom is 0.0094, 0.0088, 0.0084, 0.0078, and 0.0074 e at the electric field intensity of −0.2, −0.1, 0, 0.1, and 0.2 V/Å, respectively. In the process of transition from negative electric field to positive electric field, the decrease in the amount of electron charge obtained by the C atom means that the electrons on JSeMoSM increase, which induces the $E_{F}$ to move upward to approach the $E_{C}$ of JSeMoSM.

**Figure 6:**
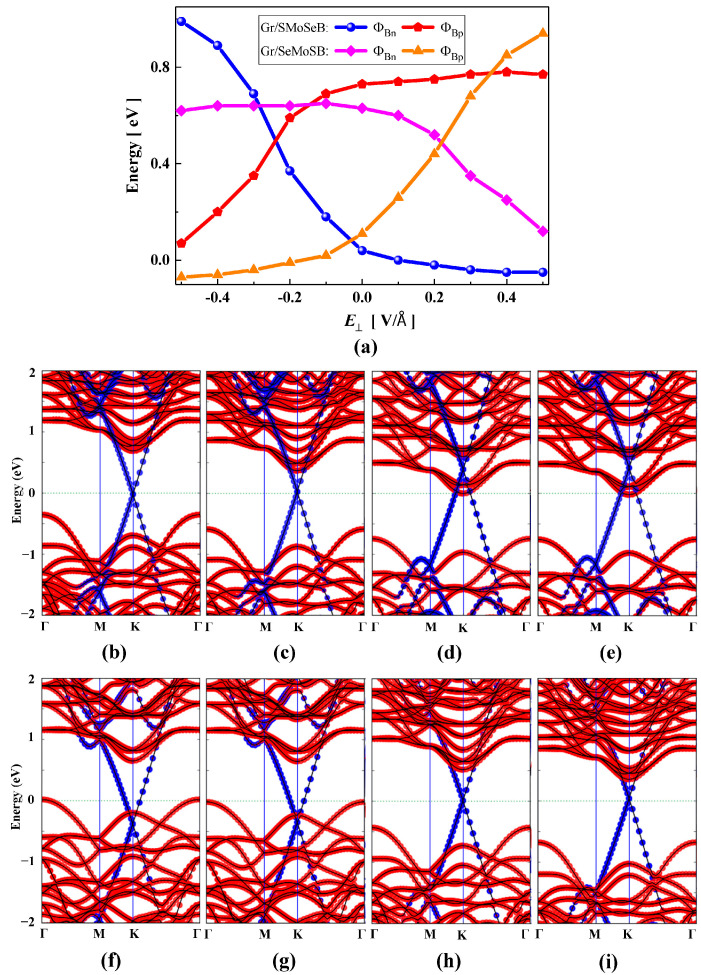
(**a**) Schottky barrier variations in Gr/JSMoSeB and Gr/JSeMoSB heterojunctions as a function of vertical electric field. The projected band structure of Gr/JSMoSeB heterojunction at the electric field strength of (**b**) −0.3, (**c**) −0.2, (**d**) 0.1, (**e**) 0.2 V/Å, respectively. The projected band structure of Gr/JSeMoSB heterojunction when the electric field strength is (**f**) −0.2, (**g**) −0.1, (**h**) 0.2, (**i**) 0.3 V/Å, respectively.

Now we turn to the study of the effect of electric fields on the Schottky barrier of Gr/JSMoSeB and Gr/JSeMoSB heterojunctions. As shown in Figure 6a, the Gr/JSMoSeB heterojunction will change from Schottky contact to Ohmic contact when the positive electric field strength is greater than 0.1 V/Å. Under a negative electric field, the n-type and p-type SBHs change in opposite directions. When the negative electric field strength is greater than −0.24 V/Å, the n-type Schottky barrier is converted into a p-type Schottky barrier. The physical mechanisms of these changes can be illustrated by the evolution of the projected band structure. Figure 6b–e describe the projected band structures of Gr/JSMoSeB heterojunction at the electric field strength of −0.3, −0.2, 0.1, and 0.2 V/Å, respectively. During the change from negative electric field to positive electric field, the redistribution of space charge induced by the electric field causes the $E_{C}$ (or $E_{V}$) of JSMoSeB to move downward relative to the $E_{F}$. As for the Gr/JSeMoSB heterojunction, when the electric field strength is greater than 0.22 V/Å, the Fermi level begins approaching the CBM, indicating an emerging n-type character, where the p-type is converted into an n-type Schottky barrier. However, Ohmic conact replaces p-type Schottky contact when the negative electric field is greater than −0.1 V/Å. The mechanism of this change can also be elucidated by the evolution of the projected band structure, which is shown in Figure 6f–i, respectively. The $E_{C}$ of JSeMoSB moves downward in a positive electric field and upward in a negative electric field.

**Figure 7:**
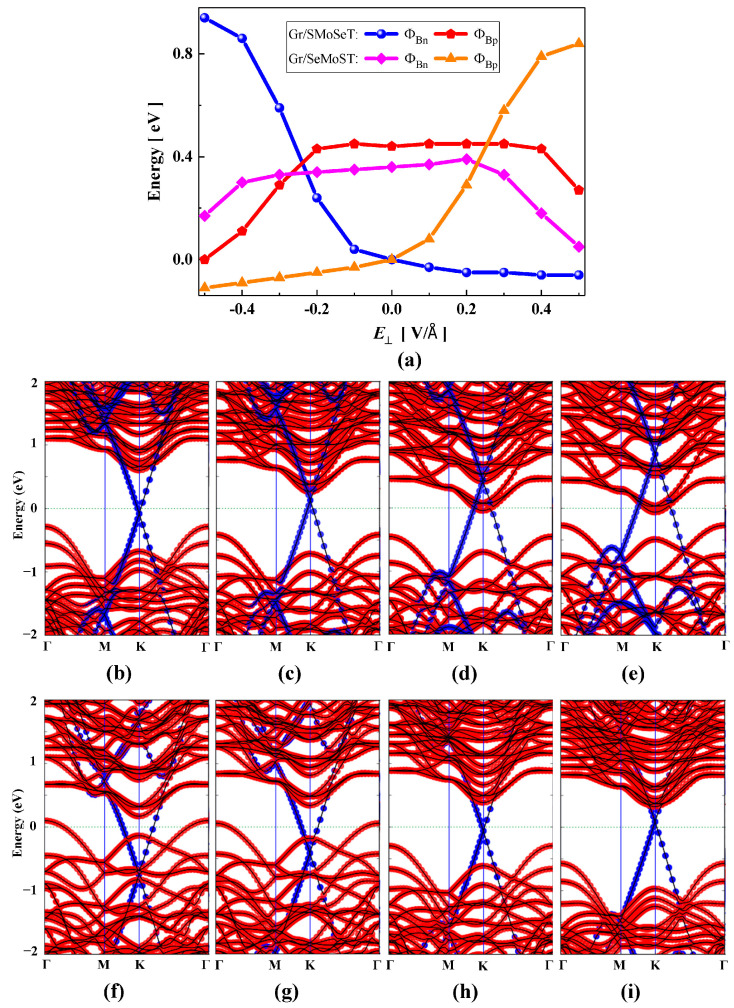
(**a**) Schottky barrier variations in Gr/JSMoSeT and Gr/JSeMoST heterojunctions as a function of vertical electric field. The projected band structure of Gr/JSMoSeT heterojunction at electric field strength of (**b**) −0.3, (**c**) −0.2, (**d**) 0.2, (**e**) 0.5 V/Å, respectively. The projected band structure of Gr/JSeMoST heterojunction when the electric field strength is (**f**) −0.5, (**g**) −0.2, (**h**) 0.2, (**i**) 0.3 V/Å, respectively.

We also studied the effect of electric fields on the Gr/JSMoSeT and Gr/JSeMoST heterojunctions. As shown in Figure 7a, the Gr/JSMoSeT heterojunction maintains an Ohmic contact under the positive electric field. However, under the negative electric field, it will be converted from Ohmic contact to n-type Schottky contact. Moreover, when the negative electric field is greater than −0.24 V/Å, the n-type SBH will be lower than the p-type SBH, which implies that the Schottky barrier type has changed. For Gr/JSeMoST heterojunction, the negative electric field keeps it in Ohmic contact. The positive electric field will change it from Ohmic contact to p-type Schottky contact. When the positive electric field strength is greater than 0.23 V/Å, the p-type will be transformed into the n-type Schottky barrier. These changes can be explained by the evolution of the projected band structure. Figure 7b–e depict the projected band structures of Gr/JSMoSeT heterojunction at the electric field strength of −0.3, −0.2, 0.2, and 0.5 V/Å, respectively. Also, Figure 7f–i exhibit the projected band structures of Gr/JSeMoST heterojunction at the electric field strength of −0.5, −0.2, 0.2, and 0.3 V/Å, respectively. The $E_{C}$ (or $E_{V}$) of JMoSSe moves downward in the positive electric field and upward in the negative electric field.

The study of the MoSSe/germanene heterojunction also indicates that the two sides of MoSSe exhibit significant differences in their contact properties, and in-plane tensile strain can induce a transition from Schottky to Ohmic contact [58]. The contact characteristics of the MoSSe/metal heterojunction can be effectively tuned by adjusting the thickness and applying biaxial strain [59]. The results obtained from these MoSSe-based heterojunctions are in good agreement with our research findings. Although the present study is theoretical, the electric field range and contact behaviors observed herein are within the reach of modern device fabrication techniques. Future experimental studies on graphene/MoSSe heterojunctions under tunable electric fields are expected to validate the theoretical predictions presented here and provide valuable guidance for the design and optimization of next-generation electronic devices.

## 4. Conclusions

In summary, the electronic properties of Gr/JMoSSe heterojunction have been systematically studied by first-principles calculations. Our results reveal that Gr interacts with JMoSSe through vdW coupling, which can preserve their intrinsic physical properties well. The different atomic planes of JMoSSe will exhibit different electronic properties when contacted with Gr. Specifically, the S atom is in contact with Gr to form an n-type Schottky barrier, whereas the contact of the Se atom with Gr forms a p-type Schottky barrier. We have found that increasing the number of layers of JMoSSe can lower the SBH of the Gr/JMoSSe heterojunctions, and the transition from the Schottky contact to the Ohmic contact can be achieved when added to the third layer. The external electric field can not only continuously adjust the SBH of the Gr/JMoSSe heterojunctions but also realize the mutual transformation of the n-type Schottky, p-type Schottky, and Ohmic contacts. The physical mechanism of the number of semiconductor layers and the electric field to control the Schottky barrier can be clarified from the transfer and redistribution of interfacial space charge. Particularly, for the Gr/JSMoSe heterojunction, increasing the number of semiconductor layers and enhancing the positive electric field strength reduces the electron charge obtained by Gr, which means that the electron charge lost on JMoSSe is reduced. Therefore, the $E_{F}$ moves downward relative to the Dirac cone of Gr and moves upward relative to the $E_{C}$ of JMoSSe. However, for the Gr/JSeMoS heterojunction, increasing the number of semiconductor layers and enhancing the negative electric field strength increases the amount of electron charge obtained by Gr, which implies that the electron charge lost on JMoSSe is increased. Hence, the $E_{F}$ moves upward relative to the Dirac cone of Gr and moves downward relative to the $E_{V}$ of JSMoSSe. Therefore, our results demonstrate that the number of semiconductor layers and the external vertical electric field can effectively control the contact characteristics of the Gr/JMoSSe heterojunction. Moreover, the fundamental physical mechanism is also systematically elaborated from the aspect of interface electron charge transfer. Our research furnishes new tactics to surmount the contact potential bottlenecks of traditional metal–semiconductor junctions and improve the performance of electronic nanodevices.

## Figures and Tables

**Table 1:** Calculated structural and electronic properties of Gr/MoSSe heterojunctions: System (*Sys*), Equilibrium Interface Spacing ($d_{\mathrm{eq}}$), Average Binding Energy ($E_{b}$), Work Function (WF), and N-type (blue) or P-type (red) Schottky Barrier Height ($\Phi_{\mathrm{Bn}(p)}$) for various layer configurations (M: monolayer, B: bilayer, T: trilayer) and terminations.

*Sys*	$d_{\mathrm{eq}}$	$E_{b}$	WF	$\Phi_{\mathrm{Bn}(p)}$
	(Å)	(meV/Å^2^ )	(eV)	(eV)
Gr	3.36	−29.78	4.46	/
JMoSSe	3.25	−27.55	4.46 (5.24)	/
Gr/JSMoSeM	3.39	−23.46	4.00	0.28
Gr/JSMoSeB	3.42	−23.77	3.76	0.04
Gr/JSMoSeT	3.48	−23.64	3.73	0
Gr/JSeMoSM	3.41	−24.54	5.45	0.61
Gr/JSeMoSB	3.40	−25.17	5.76	0.11
Gr/JSeMoST	3.43	−24.35	5.81	0

## Data Availability

The original contributions presented in the study are included in the article, further inquiries can be directed to the corresponding authors.
